# Diseased Antrum

**Published:** 1858-04

**Authors:** D. W. Roudebush


### INTERESTING CASE OF DISEASED ANTRUM.

Dr. R., having in conversation related to us his treatment and cure of a case of diseased antrum, which we believe would be of value to the profession. After considerable solicitation we prevailed upon him to give it to us, in a form for publication, as follows:—[Ed.

In May 1857, Mrs. C., of Covington, Ky., called on me, complaining of a heavy dull pain, in the left side of the upper jaw, and affecting the left eye, by causing it to twitch and jerk involuntary at times. I made a careful examination of her teeth, and found them all in a healthy condition, except the second superior bicuspid on the right side, which was badly decayed accompanied by ulceration. I advised its immediate removal, believing it to be the whole cause of her complaint, and that the pain in the opposite side of the face was sympathetic. The tooth was removed with the sac adhering to the point of the root; by the use of an astringent wash, in a few days the gum assumed a healthy condition, and the pain entirely subsided.

In the latter part of June, she returned complaining as before of pain in the left side of her face, accompanied by a twitching of the left eye. I again made a careful examination of her mouth, and finding all her teeth in a healthy condition, I at once suspected that the antrum was diseased. The first and second left superior bicuspids had been removed about a year previous, the gum was nicely healed and seemed to be in a healthy condition; but she informed me that she had been troubled six months after the teeth had been removed, before the gum finally healed. I advised her to have the gum opened, to see whether the roots of the teeth had been entirely removed, finding the absorption of the gum greater where the first bicuspid had been removed than the second. I accordingly opened the gum next the first molar, and found a rough substance, which on being removed, proved to be the remainder of the second bicuspid. With a broach I pierced the antrum, which was followed by a discharge of a thin greenish substance.

I commenced using injections of tinct. myrrh, diluted with sage tea, warm or about the temperature of blood.

On the third day a communication was formed with the left nostril, through which the lotion freely passed, and in six weeks a cure was completed. D. W. ROUDEBUSH.

